# The Relationship Between the Clinical and Radiological Findings and the Outcomes of Early Surgical Treatment After Tossy Type III Acromioclavicular Joint Dislocation

**DOI:** 10.7759/cureus.6681

**Published:** 2020-01-16

**Authors:** Povilas Masionis, Rokas Bobina, Sigitas Ryliskis

**Affiliations:** 1 Clinic of Rheumatology, Orthopaedics Traumatology and Reconstructive Surgery, Vilnius University Faculty of Medicine, Vilnius, LTU; 2 Faculty of Medicine, Vilnius University, Vilnius, LTU

**Keywords:** acromioclavicular dislocation, tossy, clavicle, rockwood

## Abstract

Background

Acromioclavicular joint (ACJ) dislocation is a common injury among young and physically active persons. Evaluating surgical outcomes clinically and radiographically is widely accepted, but it is not known which clinical tests or radiological indicators are the most important. Our hypothesis is that there is a significant correlation between clinical and radiological findings and outcomes after the early surgical treatment of Tossy type III ACJ dislocation.

Materials and methods

A retrospective study was conducted on 23 patients who underwent early surgical treatment after Tossy type III ACJ dislocation. We used the constant score (CS) and the simple shoulder test (SST) to measure the outcomes. For clinical evaluation, Paxinos, O’Brien’s, Bell-van Riet’s, and horizontal adduction tests were used. Standard A-P view radiographs were evaluated for redislocation, ACJ arthrosis, coracoclavicular (CC) space ossification, and for the presence of osteolysis.

Results

The mean time of follow-up was 16 months (range, 12-22 months). During the follow-up, all patients had one or more pathological radiological signs. All clinical tests were negative in 12 patients, seven had one positive test, three had two positive tests, and one had three positive tests. The mean CS result at the follow-up was 93.44 ± 4.90 (range, 84-100), and the mean SST result was 10.78 ± 1.51 (range, 6-12). There was no statistically significant association between the CS results and either shoulder tests or radiological findings. The SST results were statistically significantly lower for patients with positive O’Brien’s test compared to those with a negative one. In contrast, the SST results were statistically significantly higher for patients with CC space ossification, compared to those who did not have this radiological sign. Other clinical tests and radiological findings did not have any associations with the SST results.

Conclusions

We found that positive O’Brien’s test was associated with worse outcomes of early surgical treatment after Tossy type III ACJ dislocation. Despite the presence of pathological radiological signs, patients may have good or even perfect clinical outcomes after the early surgical treatment of a Tossy type III injury.

## Introduction

Acromioclavicular joint (ACJ) dislocation is a common injury among young people between 20 and 40 years old who practice sports, and it has an incidence of between 9% and 17% of all shoulder injuries [1-6]. The pattern of this trauma is a direct force to the acromion with the arm in an adducted position [7]. ACJ dislocations were classified for the first time in 1963 by Tossy from type I to type III according to the injury pattern of the acromioclavicular (AC) and coracoclavicular (CC) ligaments [8,9]. Later, the classification was expanded by Rockwood and Green to types I to VI [4,9-11]. It is widely accepted that type I-II injuries should be treated conservatively, and type IV-VI surgically [3-5,12-15]. The treatment of Rockwood type III injuries remains an object for debate: some studies advocate surgical treatment whilst others do not see a significant difference between surgical and conservative approaches, and highlight that each case should be treated individually [13,14,16]. At the time of writing this article no single surgical technique is accepted as a standard for the treatment of ACJ dislocation. Moreover, there are more than 100 surgical procedures described in the literature which makes it more difficult to compare clinical results [16]. There is evidence in the literature that clinical tests are a valuable tool to investigate the ACJ pathology [17,18]. However, it is not known which of the clinical signs are associated with worse outcomes after the early surgical treatment of ACJ dislocation.

The aim of the present study is to investigate which clinical and radiological findings are the most important for evaluating the outcomes of early surgical treatment after Tossy type III ACJ dislocation. Our hypothesis is that there is a significant correlation between clinical and radiological findings and outcomes after early surgical treatment.

## Materials and methods

Patients and clinical assessment

Over a one-year period, 29 patients with acute complete ACJ dislocation were admitted to the Republican Vilnius University Hospital in Vilnius, Lithuania, and were included in this retrospective study after the approval of the Ethics committee of the hospital. Informed consent was obtained from each study participant. All of the patients had a Tossy type III injury and underwent open ACJ fixation. The characteristics of the Tossy and Rockwood classifications are presented in Table 1.

**Table 1:** The characteristics of the Tossy and Rockwood classifications. AC, acromioclavicular; CC, coracoclavicular; DT, deltotrapezoidal [4,8-11]

Type of injury		AC capsule	CC ligament	DT fascia	Clavicle position
Tossy	Rockwood				
I	I	Sprained	Intact	Intact	Not displaced
II	II	Torn	Sprained	Intact	<25% superior its thickness
III	III	Torn	Torn	Injured	25%-100% superior
	IV	Torn	Torn	Detached	Posterior to acromion
	V	Torn	Torn	Detached	>100% superior
	VI (very rare)	Torn	Torn	Detached	Under coracoid

In all cases, the time between trauma and surgery was less than three weeks. In cases of ACJ fixation with metal implants, all patients were investigated after the hardware had been removed.

Of the 29 patients, 23 (79%, 22 men and one woman) participated in the clinical and radiological follow-up, as two patients refused to participate in the follow-up and four patients were out of reach and could not be contacted. The mean follow-up time after the initial injury was 16 months (range, 12-22 months). ACJ was remedied by suture loop in 16 cases, by hook plate in two cases, and by tension band wire in five cases. The surgeries were performed by two experienced surgeons who are not co-authors of the present study. Each patient was invited to evaluate the outcomes of these procedures using the constant score (CS, maximal result - 100) and the simple shoulder test (SST, maximal result - 12) questionnaires. A digital dynamometer (Kern & Sohn GmbH*, *Balingen, Germany) was used to evaluate the abduction strength of operated and healthy shoulders. Four shoulder tests were used for clinical evaluation: horizontal adduction, O’Brien’s, Paxinos, and Bell-van Riet’s [17,18]. The horizontal adduction test is performed by the examiner, who stands behind the patient on the side being tested. The examiner then grasps the patient’s arm at an elbow in a slightly distal position, passively flexes the patient’s shoulder to 90 degrees, and then maximally adducts the patient’s shoulder (bringing it across their body towards the other shoulder). For O’Brien’s test, the arm to be tested should be in 90 degrees of flexion and approximately 10 degrees of adduction. The patient then internally rotates the arm, pronating at the elbow and essentially pointing the thumb to the ground. The examiner provides a downward force distally on the arm, while the patient resists with an upward force. Bell-van Riet’s test is performed similarly to O’Brien’s test, with the key difference being that the patient’s arm is in full adduction (as far as possible across the chest). The Paxinos test is performed with the patient sitting with the symptomatic arm by their side. The examiner's thumb is then placed under the posterolateral aspect of the acromion, and the index and middle fingers of the same (or contralateral) hand are placed superiorly to the mid-clavicle. Pressure is then applied to the acromion in an anterosuperior direction with the thumb, while also applying pressure in an inferior direction to the mid-clavicle with the index and middle fingers. All of the above described tests are considered to be positive if pain is provoked. 

Radiographic examination

**Figure 1:**
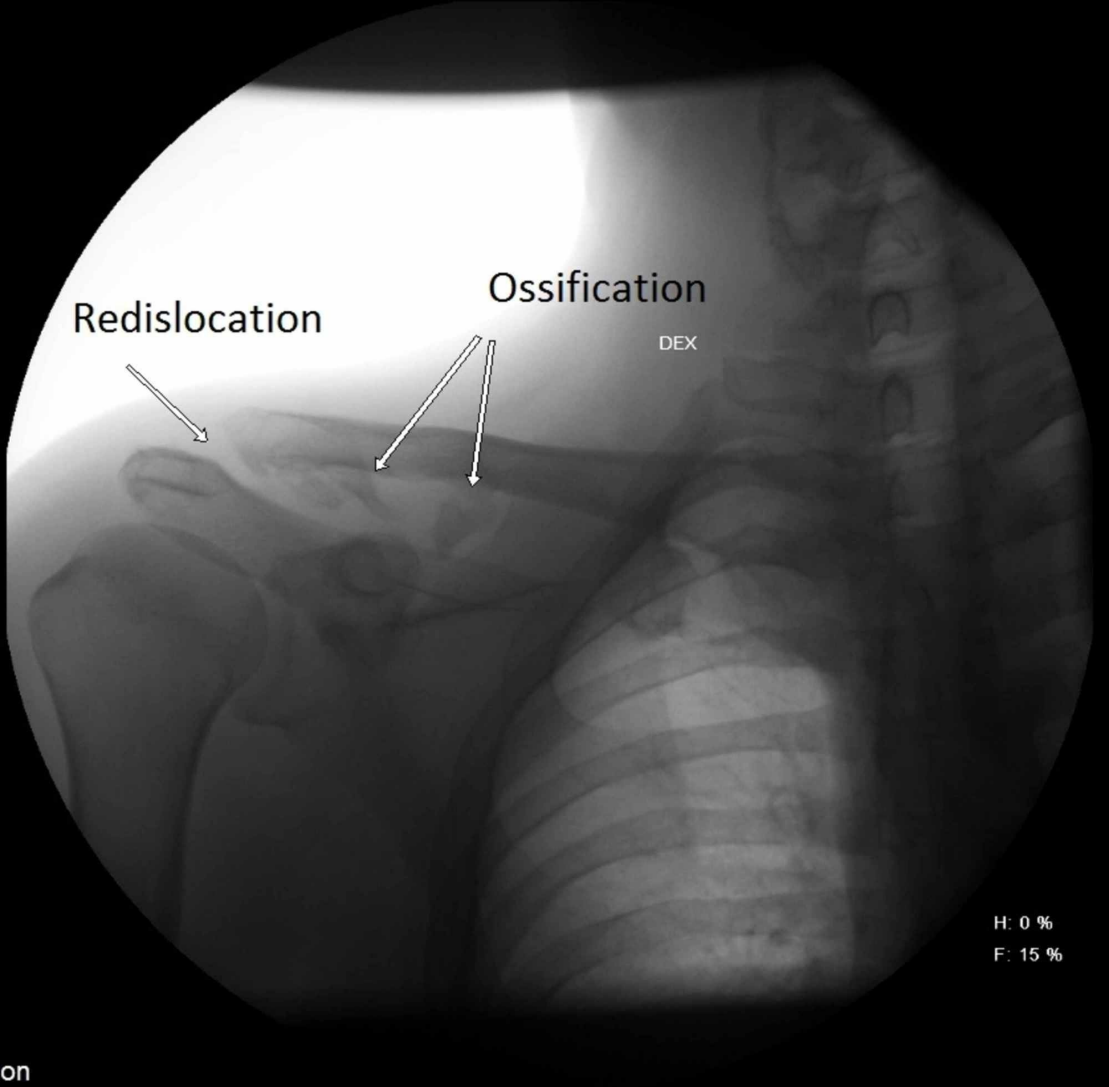
A radiograph of a 40-year-old male patient 20 months after ACJ reconstruction with suture loop. Recurrent dislocation of the clavicle and CC space ossification are present. ACJ, acromioclavicular joint; CC, coracoclavicular

**Figure 2:**
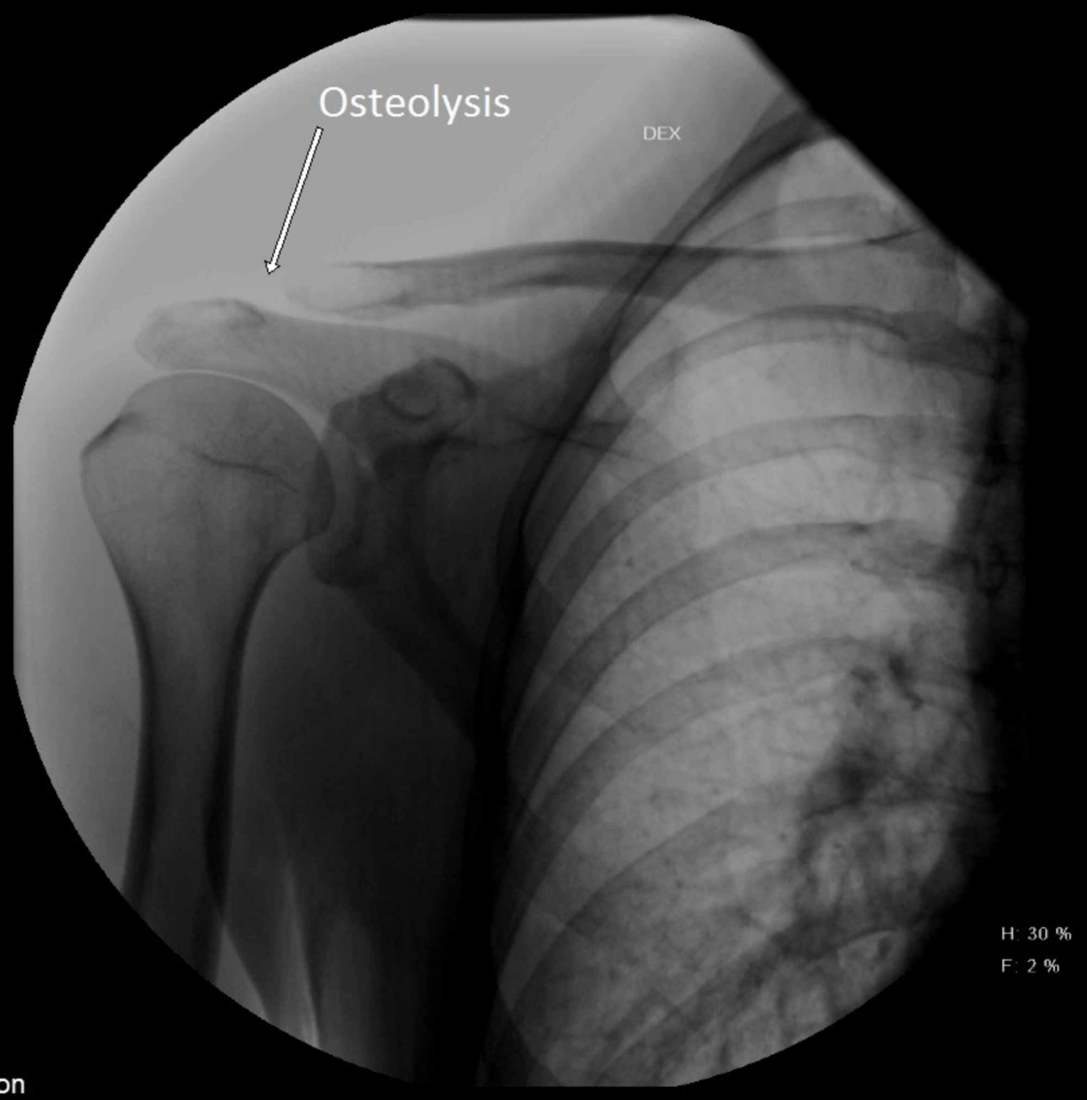
A radiograph of a 31-year-old male patient 16 months after ACJ reconstruction with suture loop. Osteolysis of the acromial end of the clavicle is present. ACJ, acromioclavicular joint

Each patient underwent a postoperative radiographic examination. Standard A-P radiograms were performed and evaluated for recurrent dislocation (according to Tossy), osteolysis of the acromial end of the clavicle, ossification of the CC space, and ACJ arthrosis (Figures 1, 2).

 

Statistical analysis

IBM SPSS Statistics v23.0 (IBM Corp., Armonk, NY) software was used for statistical analysis. MS Excel software was used for additional calculations. The Shapiro-Wilk test was used to assess the normal distribution of SST and CS results. For the comparison between groups, the two tests were employed. Student’s t-test was used for parametric variables, and the Mann-Whitney U test was used for non-parametric variables.

## Results

Of the 23 patients that participated, 22 (96%) were male and one (4%) was female. The mean age was 35.43 ± 10.45 years. During the follow-up, all patients had one or more radiological signs. Eight patients had Tossy type I dislocation, seven patients had Tossy type II, and one patient had Tossy type III. Additionally, 11 had signs of ACJ arthrosis, in seven cases osteolysis of the distal clavicle was present, and six patients had radiological signs of CC space ossification (Table 2).

**Table 2:** The radiological findings and data regarding the patients. "+" stands for present and "-" for absent ACJ, acromioclavicular joint; CC, coracoclavicular

Age	Fixation technique	Tossy	ACJ arthrosis	Osteolysis of the distal clavicle	Ossification of the CC space
30	Suture loop	I	-	+	-
31	Tension band wire	I	+	+	-
34	Tension band wire	I	+	-	-
23	Suture loop	I	-	-	-
33	Suture loop	-	-	+	-
34	Suture loop	II	+	-	+
30	Hook plate	-	-	+	-
29	Suture loop	II	-	-	-
50	Hook plate	II	+	+	-
40	Suture loop	III	+	-	+
53	Suture loop	-	-	-	-
28	Suture loop	-	-	+	-
22	Suture loop	-	-	-	-
52	Suture loop	I	-	-	-
28	Suture loop	II	+	-	+
51	Suture loop	I	+	-	-
48	Tension band wire	II	-	-	-
37	Suture loop	II	+	-	-
23	Suture loop	I	+	-	+
52	Tension band wire	II	+	-	+
23	Suture loop	-	+	-	-
31	Tension band wire	I	-	-	-
33	Suture loop	-	-	+	+

All clinical shoulder tests were negative in 12 patients, seven had one positive test, three had two positive tests, and one had three positive tests. The mean CS result at the follow-up was 93.44 ± 4.90 (range, 84-100), and the mean SST result was 10.78 ± 1.51 (range, 6-12). The mean abduction power in a healthy shoulder was 11.85 ± 1.81 kg (range, 7.88-15.00 kg), compared to 10.37 ± 2.11 kg (range, 6.17-14.90) in injured shoulder. There were no statistically significant differences in CS results between positive and negative clinical shoulder tests and radiological findings (Table 3).

**Table 3:** A comparison of CS results between positive and negative clinical tests and radiological symptoms. SD, standard deviation; CC, coracoclavicular; ACJ, acromioclavicular joint

Questionnaire	Evaluation	Test or symptom	Negative (mean ± SD)	Positive (mean ± SD)	p-value
Constant score (CS)	Clinical evaluation	Horizontal adduction	94.05 ± 4.71	89.33 ± 4.93	0.122
		Bell-van Riet’s	94.35 ± 4.86	90.83 ± 4.36	0.133
		Paxinos	94.16 ± 5.05	90.00 ± 2.00	0.125
		O’Brien’s	93.75 ± 4.96	91.33 ± 4.73	0.438
	Radiological evaluation	Osteolysis of the distal end of clavicle	93.69 ± 4.92	92.86 ± 5.18	0.717
		Ossification of CC space	91.56 ± 3.64	94.64 ± 5.33	0.144
		ACJ arthrosis	93.73 ± 4.65	93.17 ± 5.31	0.561

SST results were statistically significantly lower for patients with positive O’Brien’s test compared to a negative one (8.33 ± 2.52 vs. 11.15 ± 0.93, respectively, p = 0.046). In contrast, SST results were statistically significantly higher for patients with CC space ossification compared to those who did not exhibit this radiological sign (11.29 ± 1.07 vs. 10.00 ± 1.80, respectively, p = 0.016). The differences of SST results between other positive and negative clinical shoulder tests and radiological findings were not statistically significant. More detailed results are presented in Table 4.

**Table 4:** A comparison of SST results between positive and negative clinical tests and radiological symptoms. SD, standard deviation; CC, coracoclavicular; ACJ, acromioclavicular joint

Questionnaire	Evaluation	Test or symptom	Negative (mean ± SD)	Positive (mean ± SD)	p-value
Simple shoulder test (SST)	Clinical test	Horizontal adduction	11.00 ± 1.17	9.33 ± 2.89	0.230
		Bell-van Riet’s	11.06 ± 1.43	10.00 ± 1.55	0.087
		Paxinos	10.63 ± 1.61	11.50 ± 0.58	0.324
		O’Brien’s	11.15 ± 0.93	8.33 ± 2.52	0.046
	Radiological sign	Osteolysis of the distal end of clavicle	11.13 ± 0.96	10.00 ± 2.24	0.308
		Ossification of CC space	10.00 ± 1.80	11.29 ± 1.07	0.016
		ACJ arthrosis	10.55 ± 1.86	11.00 ± 1.13	0.740

The differences of both CS and SST results between Tossy types at the time of the follow-up were not statistically significant. 

## Discussion

To the best of our knowledge, this is the first report about the relationship between specific shoulder tests and the outcomes of operative treatment after Tossy type III ACJ dislocation. The present study showed that positive O’Brien’s test was associated with worse outcomes in SST scores only, while other clinical shoulder tests did not reveal such an association. Moreover, the presence of CC space ossification was associated with better outcomes in SST scores, whereas the presence of other pathological radiological signs was not correlated with significant changes in outcomes. In fact, despite the presence of pathological radiological signs patients may have good or even perfect outcomes after early surgical treatment. This finding is consistent with similar phenomena described in the literature (Table 5).

**Table 5:** A comparison of the data of different studies found in the literature. SD, standard deviation; NA, not available

Study and year	Sample size	Mean age (years)	Mean follow-up time (months)	Simple shoulder test (SST) results: mean ± SD	Constant score (CS) results: mean ± SD	Rate of redislocation
Present study	23	35.43	16	10.78 ± 1.51	93.44 ± 4.90	69%
Motta et al. 2012 [4]	34	36	65	11 ± 1	97 ± 6	39%
Tienen et al. 2003 [19]	21	33	36	NA	97 (range, 66-100)	14%
Ejam et al. 2008 [20]	6	40	26	NA	97 (range, 92-100)	0%
Lim et al. 2007 [6]	7	35	6	NA	NA	50%
Rolf et al. 2008 [21]	29	37	54	NA	87	69%

Furthermore, the presence of CC space ossification was found to increase the stiffness of the surgical reconstruction, and is associated with better clinical outcomes as higher grade ossification possibly increases the stiffness of ACJ fixation [4,15,22]. The same phenomenon was observed in the present study. However, the influence of redislocation and osteolysis of the acromial end of the clavicle on the treatment outcomes remains unclear.

From the biomechanical aspect, all described tests produce direct or indirect compression to the ACJ, which causes pain in unhealthy joint. Only Paxinos test is based on direct compression by pushing the patient’s acromion in the anterosuperior direction with the thumb, and the clavicle in the posteroinferior direction with the index and middle fingers. Furthermore, there is evidence in the literature that a positive Paxinos test combined with a positive bone scan is the best predictor for ACJ pathology [17]. However, we did not find this test to be associated with worse outcomes after ACJ fixation. Horizontal adduction, O‘Brien’s, and Bell-van Riet’s tests are based on indirect compression to the ACJ. The scapula is driven or rotated towards the midline by the horizontal adduction of the arm in the horizontal adduction test, and O‘Brien’s and Bell-van Riet’s tests additionally involve internal rotation and forced elevation, which creates an additional compression through the supraspinatus muscle. Since O‘Brien’s and Bell-van Riet’s tests have an additional compression, these tests have been found to have a high sensitivity to ACJ pathology [18]. However, we found that only O‘Brien’s test was associated with worse treatment outcomes.

Our institution gives priority to the surgical treatment of Rockwood type III injury for younger and physically active individuals or manual labor workers. Considering that Tossy III consists of Rockwood III-VI and the majority of our patients are young and active, in clinical work we prefer the Tossy classification. It would probably be more beneficial to use the Rockwood classification in clinical work because recent literature defines the Rockwood classification as a gold standard for grading ACJ dislocations, and shows excellent inter- and intra-observer reliability for acute ACJ dislocations [11]. 

There is a wide spectrum of opinions regarding the point in time that defines early and delayed surgical treatment [4,20,21,23-27]. The dividing line has been described across the literature as two, three, four, or even six weeks after the injury. Song et al. performed a literature review of acute and delayed surgical treatment outcomes and concluded that three weeks is the most clinically relevant dividing line, as acute pain generally disappears between two and three weeks after the ACJ dislocation [15]. We believe that the three-week point provides a solid foundation for further research. Were future studies to proceed on this basis, the comparability of all further work would be greatly enhanced as all references to "early" or "delayed" would refer to the same period of time.

There are some limitations of the present study. To begin, three fixation techniques were used: tensions band wire, CC interval fixation by suture loops, and hook plate. Furthermore, the surgical procedures were performed by two different surgeons. Both factors potentially had an influence on clinical outcomes. On the other hand, we did not aim to evaluate different reconstruction techniques or the performances of different surgeons. The main interest of this study was the evaluation of post-traumatic sequelae of the ACJ.

## Conclusions

We found that positive O’Brien’s test was associated with worse treatment outcomes of early surgical treatment after Tossy type III ACJ dislocation. Despite the presence of pathological radiological signs, patients may have good or even perfect clinical outcomes after early surgical treatment of Tossy type III injury.
